# Supplementary material for: CENP-E activation by Aurora A and B controls kinetochore fibrous corona disassembly
Source: Nat Commun. 2023 Sep 1;14:5317. doi: 10.1038/s41467-023-41091-2 (PMC10474297; doi:10.1038/s41467-023-41091-2)
Supplement: Supplementary file 1 — Supplementary Information [file 41467_2023_41091_MOESM1_ESM.pdf]

CENP-E activation by Aurora A and B controls kinetochore fibrous corona disassembly

Supplementary Information

Susana Eibes[1], Girish Rajendraprasad[1], Claudia Guasch-Boldu[1], Mirela Kubat[1], Yulia Steblyanko[1] and Marin Barisic[1,2*]

[1] Cell Division and Cytoskeleton, Danish Cancer Society Research Center, Copenhagen, Denmark

[2] Department of Cellular and Molecular Medicine, Faculty of Health and Medical Sciences, University of Copenhagen, Copenhagen, Denmark

[*] Correspondence to: barisic@cancer.dk

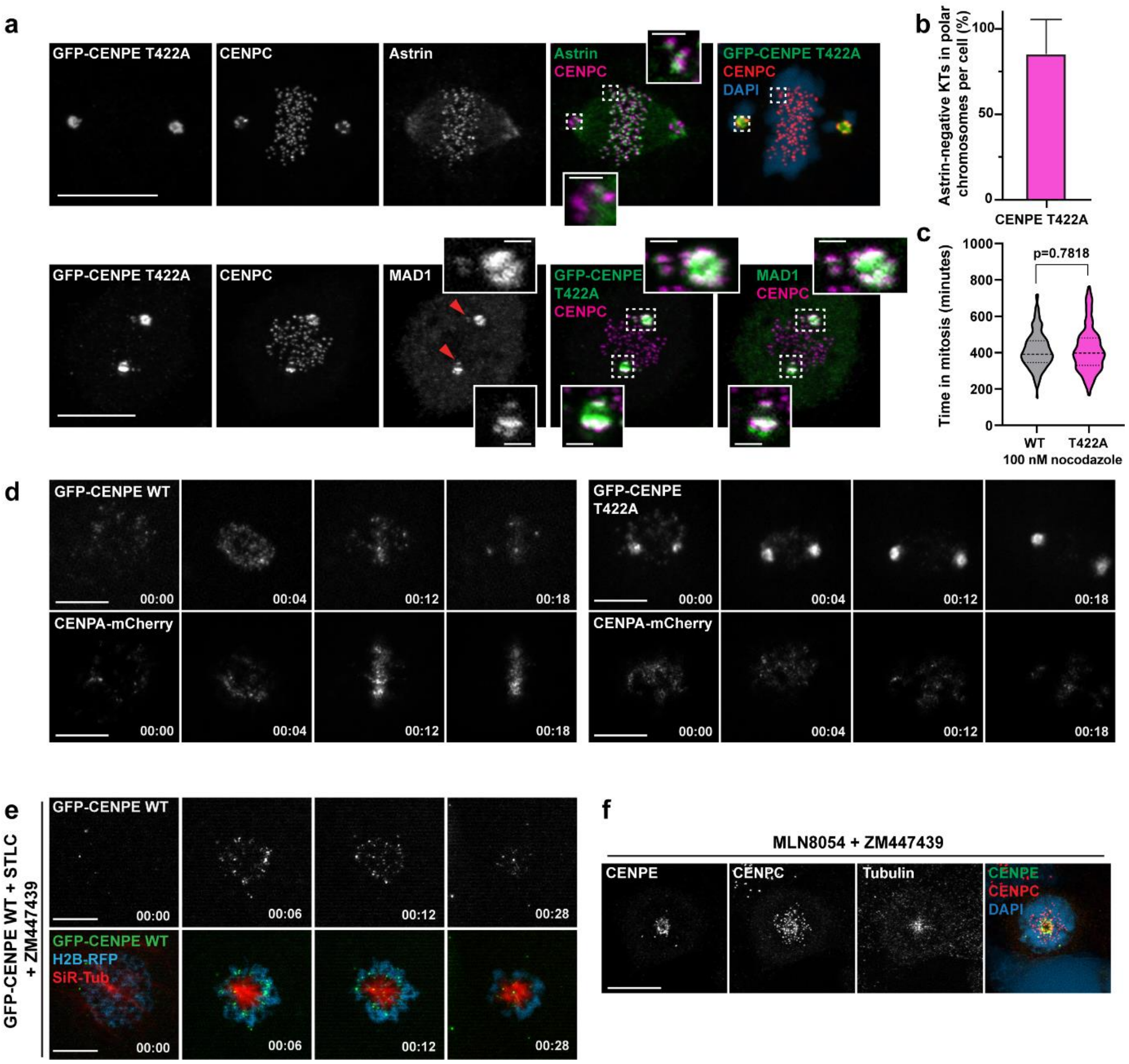

Supplementary Fig. 1. Related to Figure 1. (a) Representative maximum intensity-projected point-scanning confocal images of astrin and MAD1 immunostaining in U2OS GFP-CENP-E T422A cells arrested in pseudo-metaphase. (b) Quantification of the percentage of astrin-negative polar chromosomes per cell. Values are plotted with mean ± SD. N (cells) = 35; N (independent experiments) = 3. Scale bar: 10 µm; scale bar in insets: 1µm. (c) SAC robustness assay measuring the time in mitosis of WT and T422A cells treated with 100 nM nocodazole. Violin plots with median (thick dashed lines) and quartiles (light dashed lines) are presented. N (number of cells, number of independent experiments): WT (152, 3), T422A (152, 3). Statistical significance was determined by the Mann-Whitney U-test (unpaired, two-tailed; no normal distribution). p values are indicated. (d) Representative spinning-disk confocal images of CENP-A-mCherry-expressing GFP-CENP-E WT and T422A U2OS cells used for

quantification of GFP-CENP-E dynamics at KTs. Scale bar: 10 μm. Time: hour:min. (e) Representative spinning-disk confocal time-series of GFP-CENP-E WT monopoles treated with ZM447439 inhibitor. Time scale: hour:min. Scale bar: 10 μm. (f) Representative maximum intensity-projected point-scanning confocal images of U2OS parental cell line monopoles treated with Aurora A and B inhibitors simultaneously immunostained against CENP-E, CENP-C and α-tubulin. Scale bar: 10 μm.

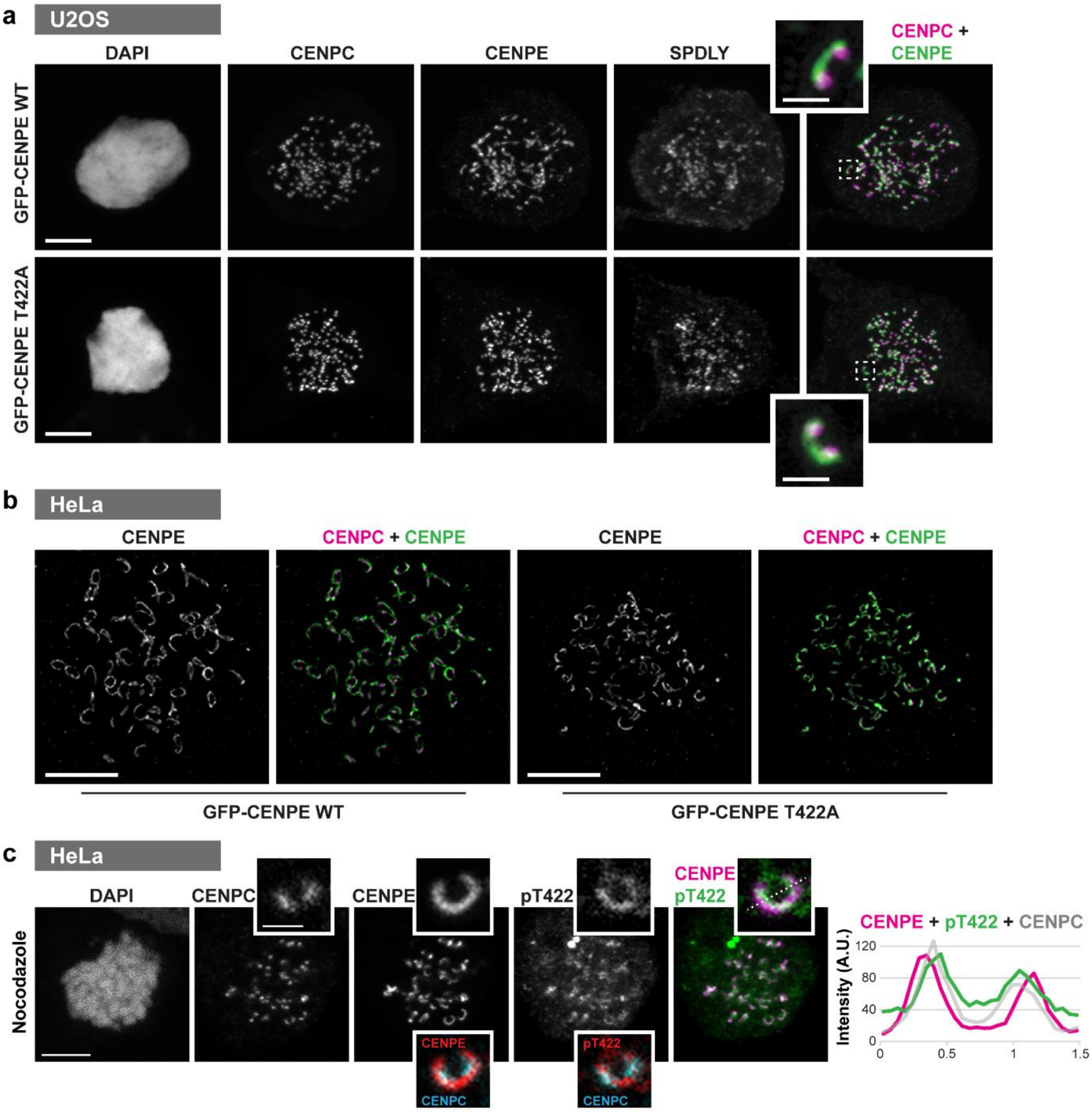

Supplementary Fig. 2. Related to Figure 2. (a) Representative maximum intensity-projected point-scanning confocal images of expanded coronas in nocodazole treated U2OS GFP-CENP-E WT/T422A cell lines. Scale bar: 5 µm; scale bar in insets: 1µm. (b) Representative lattice structured illumination microscopy (SIM) images of fully expanded coronas in HeLa cells transfected with GFP-CENP-E WT and 422A, used for the quantification of corona volume in Fig. 2g. Scale bar: 5 µm. (c) Representative maximum intensity-projected point-scanning confocal images of phosphorylated CENP-E (pT422) localization in nocodazole-treated HeLa cells, together with the intensity profile of the region represented by a white dashed line. Scale bar: 10 µm.

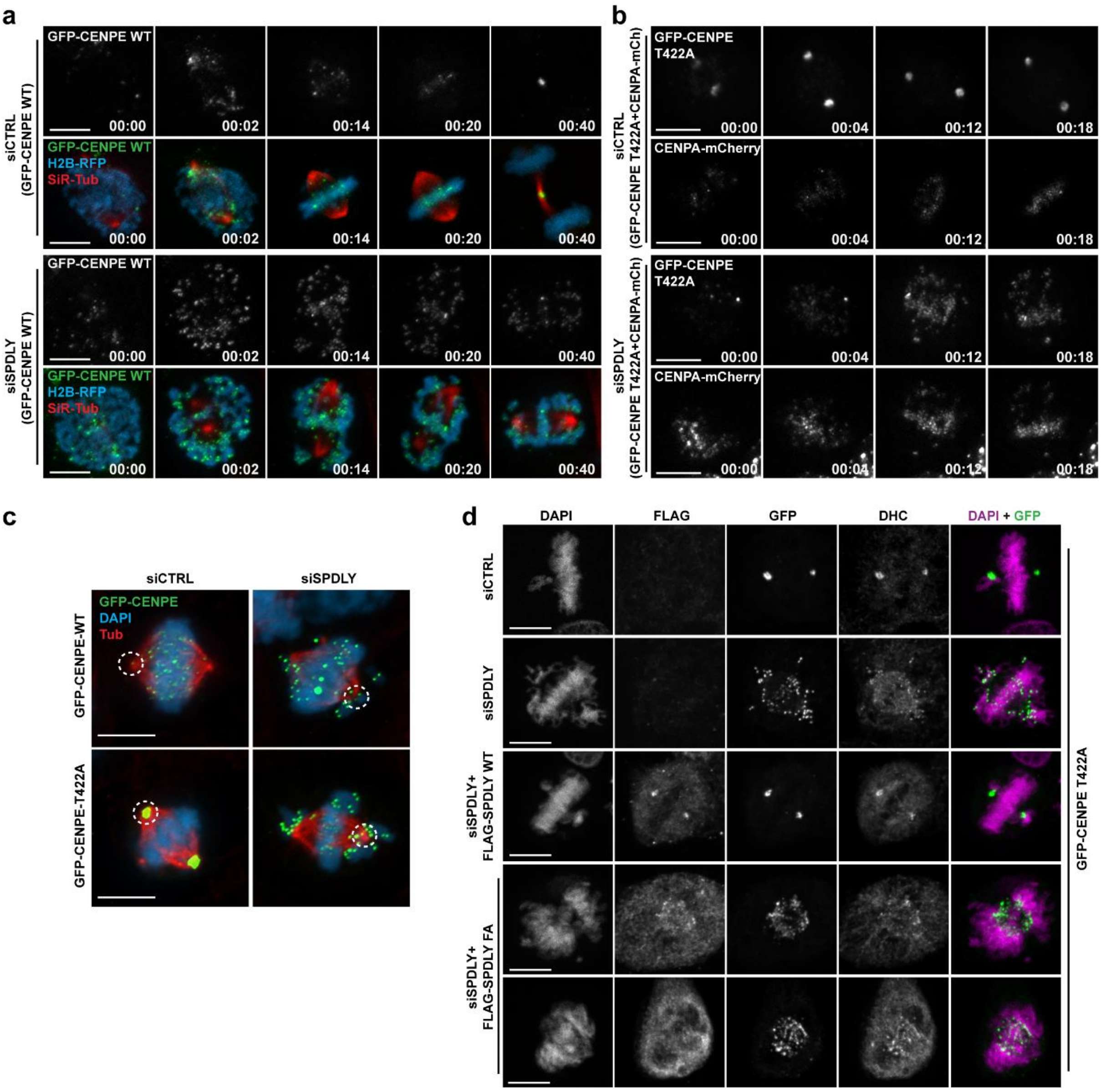

Supplementary Fig. 3. Related to Figure 3. (a) Spinning-disk confocal time-series of mitosis in U2OS GFP-CENP-E WT cells transfected with siCTRL or siSPDLY. Scale bar: 10 µm. Time scale: hour:min. (b) Spinning-disk confocal time-series of CENP-A-mCherry-expressing GFP-CENP-E T422A U2OS cells transfected with siCTRL or siSPDLY, used for quantification of GFP-CENP-E dynamics at KTs. Scale bar: 10 µm. Time scale: hour:min. (c) Representative spinning-disk confocal images of immunostainings of U2OS WT/T422A cells for spindle pole intensity quantification. The cells were immunostained with antibodies against GFP and α-tubulin, with DAPI as DNA counterstain. Scale bar: 10 µm. (d) Representative spinning-disk confocal images of GFP-CENP-E T422A cells undergoing indicated transfections and immunostained using target specific antibodies, with DAPI as DNA counterstain (quantified in Fig. 3d, e). Scale bar: 10 µm.

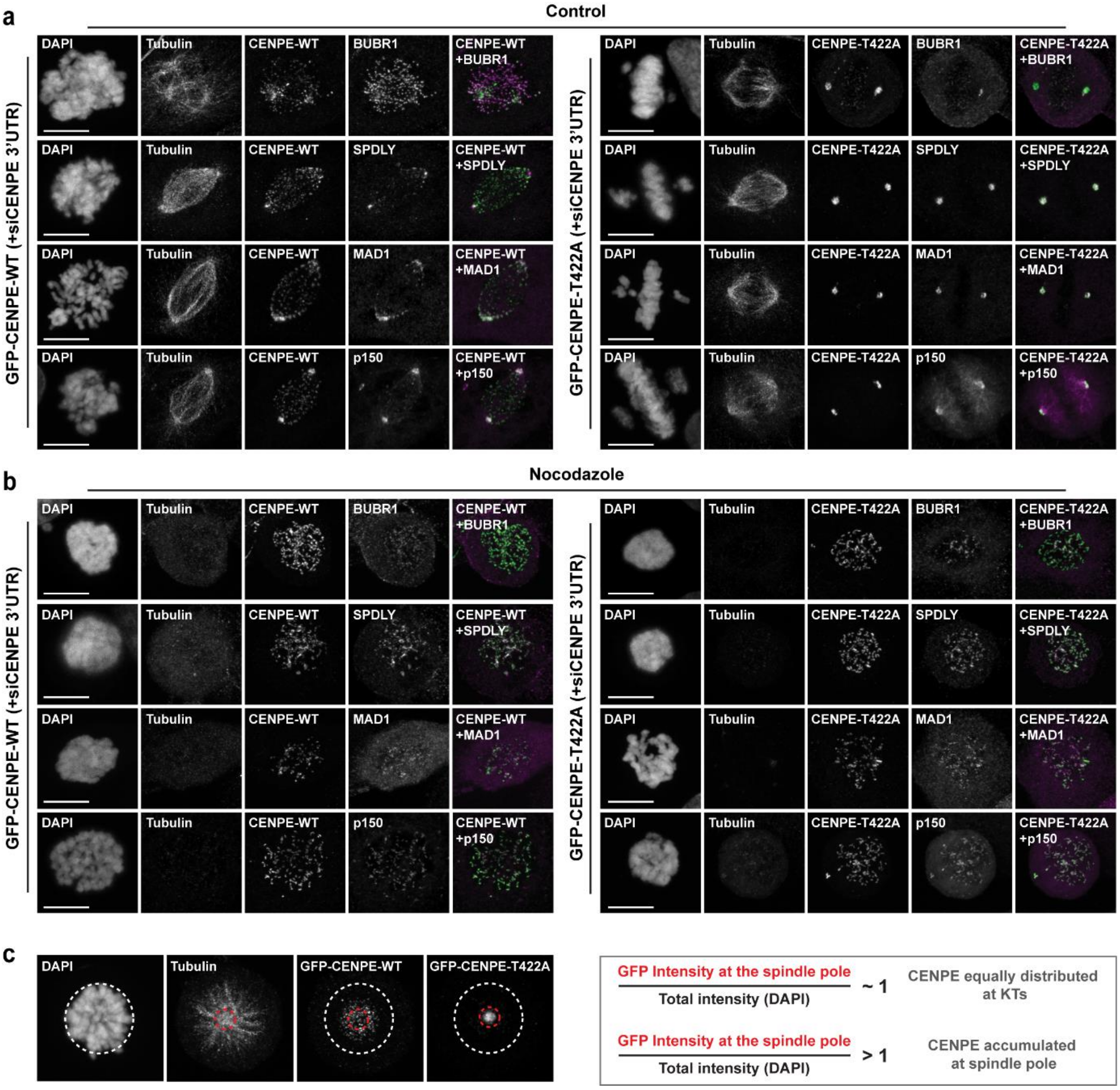

Supplementary Fig. 4. Related to figure 4. (a) Representative maximum intensity-projected point-scanning confocal images of immunostainings of the indicated proteins in U2OS GFP-CENP-E WT/T422A cell lines in prometaphase/pseudo-metaphase. Scale bar: 10 µm. (b) Representative maximum intensity-projected point-scanning confocal images of immunostaining for the indicated proteins in U2OS GFP-CENP-E WT/T422A cells treated with nocodazole. Scale bar: 10 µm. (c) Schematic representation of the method used for quantification of spindle pole accumulation of corona proteins in monopoles (quantified in Fig. 4e and Fig. 5d). A circular ROI covering all the chromosomes was used to measure the total mean intensity of the selected protein and a smaller circular ROI, was used to measure the mean intensity at the spindle pole. DAPI and tubulin served as references for the big and small circular ROI, respectively. Spindle pole intensity was normalized to the total intensity, thus, a

ratio equal to 1 indicates equal distribution of the protein, whereas a ratio higher than 1 indicates accumulation of the protein in the smaller area.

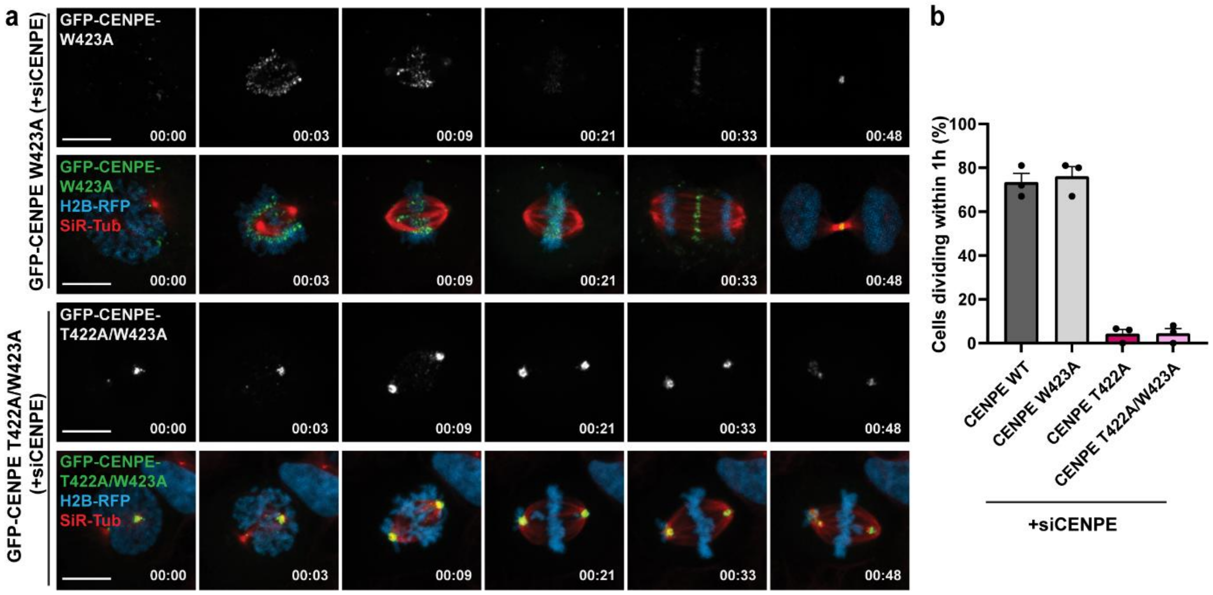

Supplementary Fig. 5. Related to figure 5. (a) Spinning-disk confocal time-series of mitosis in U2OS cells with inducible expression of GFP-CENP-E W423A and GFP-CENP-E T422A/W423A. H2B-RFP and SiR-tubulin used to visualize DNA and MTs, respectively. Time scale: hour:min. Scale bar 10 µm. (b) Quantification of the percentage of cells exiting mitosis within one hour in the mentioned cell lines (right). Values are plotted with mean ± SEM. N (number of cells, number of experiments) WT (29, 3), W423A (48, 3), T422A (40, 3), T422A/W423A (39, 3).

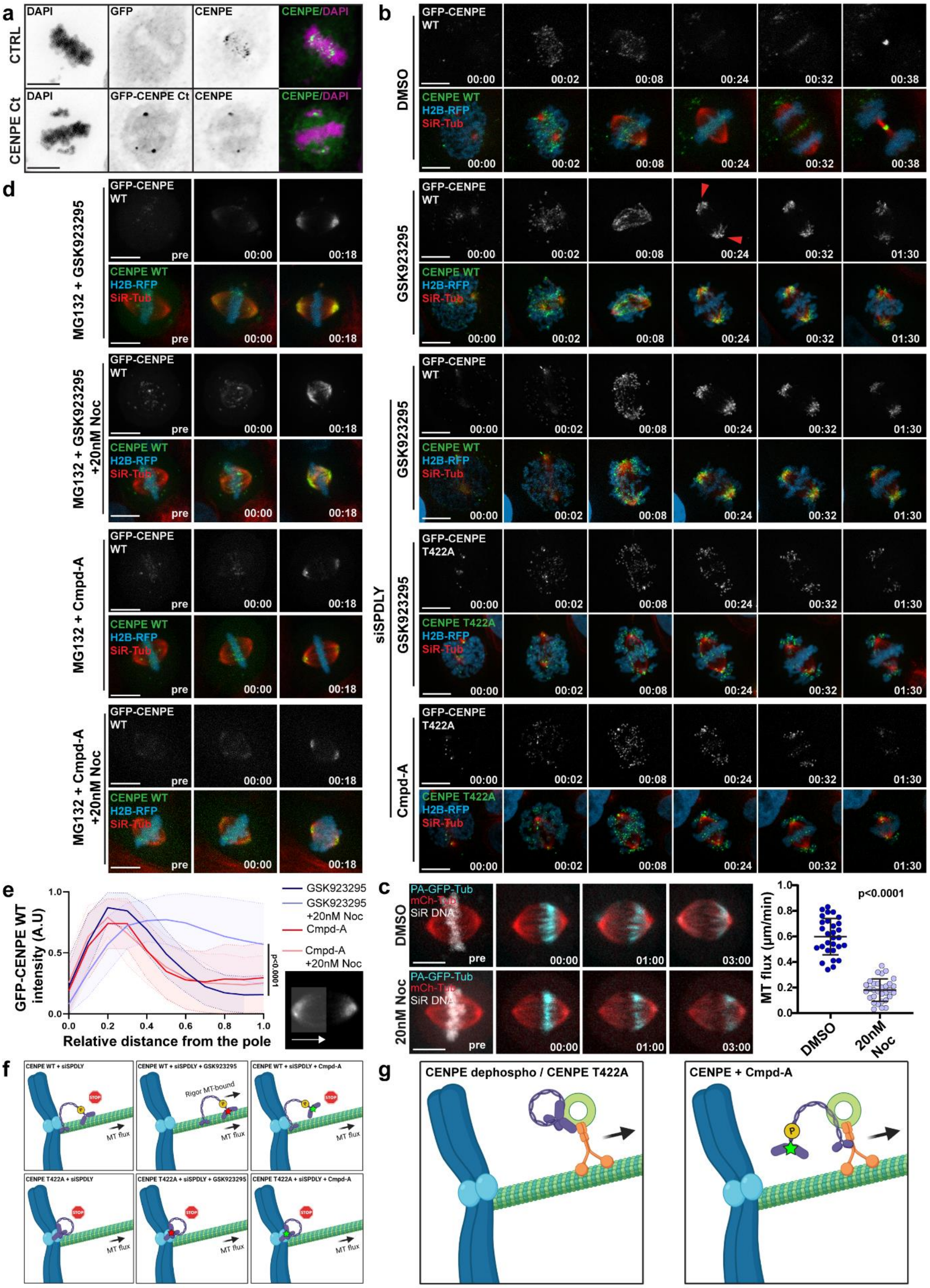

Supplementary Fig. 6. Related to figure 5. (a) Representative spinning-disk confocal images of U2OS cells transfected with an empty vector or FLAG-GFP-CENP-E (1779-2701). Scale

bar: 10 μm. (b) Representative spinning-disk confocal time-series of mitosis in U2OS GFP-CENP-E WT/T422A cells following the indicated treatments. Time: hour:min. Scale bar: 10 μm. (c) Left: Representative spinning-disk confocal time-lapse images showing MT flux in U2OS PA-GFP/mCherry-α-tubulin cells following the indicated treatments. DNA counterstained by SiR-DNA is shown in gray, photoactivated MTs in cyan, and mCherry-α-tubulin in red. Time: min:sec. Scale bar: 10 μm. Right: MT flux rates of metaphase-arrested spindles with indicated treatments shown as scatter plot with their mean and SD (n = 29 cells for DMSO, and 31 cells for 20 nM nocodazole from three independent experiments). Statistical significance was determined by the Mann-Whitney U-test (unpaired, two-tailed; no normal distribution). p values are indicated. (d) Representative spinning-disk confocal time-series of MG132-arrested U2OS GFP-CENP-E WT cells treated with the indicated CENP-E inhibitors in control or MT flux-depleted conditions. Images from before and after the addition of inhibitors are shown. Time: hour:min. Scale bar: 10 μm. (e) Normalized GFP-CENP-E WT intensity profiles of half-spindles for the indicated treatments. Solid line follows the mean, dotted lines with shades represent the SD. N (number of cells, number of independent experiments): GSK923295 (23, 3), GSK923295 + 20 nM Noc (28, 3), Cmpd-A (25, 3), Cmpd-A + 20 nM Noc (24, 3). Statistical significance was determined by the Mann-Whitney U-test (unpaired, two-tailed; no normal distribution). p values are indicated. (f) Illustrated model of the effect of CENP-E inhibitors on CENPE WT/T422A localization in the absence of dynein-mediated stripping (siSPDLY). GSK923295-inhibited CENP-E is rigor-bound to MTs and therefore accumulates at the spindle poles via MT flux. Cmpd-A-induced spindle pole accumulation of CENP-E depends exclusively on dynein-mediated stripping. Folded conformation of CENP-E T422A prevents its rigor-binding to MTs upon GSK932295-mediated inhibition. Therefore, CENP-E T422A cannot be transported to the poles via MT flux, and its spindle pole accumulation exclusively depends on dynein-mediated stripping. (g) Illustrated model comparing the effect of CENP-E inhibition/dephosphorylation on premature stripping of the fibrous corona. Illustrations created with BioRender.com.
