# Supplementary material for: CENP-E activation by Aurora A and B controls kinetochore fibrous corona disassembly
Source: Nat Commun. 2023 Sep 1;14:5317. doi: 10.1038/s41467-023-41091-2 (PMC10474297; doi:10.1038/s41467-023-41091-2)
Supplement: Supplementary file 3 — Description of Additional Supplementary Files [file 41467_2023_41091_MOESM3_ESM.pdf]

Description of Additional Supplementary Files

File name: Supplementary Data 1
Description: Numerical values of the represented data.

File name: Supplementary Movie 1
Description: Mitosis in cells expressing GFP-CENP-E T422A. Spinning-disk confocal live-cell imaging of stable U2OS cell lines with inducible expression of GFP-CENP- E WT or T422A (grey and green), infected with AV-H2B-RFP for DNA visualization (blue) and tubulin stained using SiR-tubulin (red). Time: hour:min.

File name: Supplementary Movie 2
Description: CENP-E localization upon Aurora A inhibition in STLC- induced monopolar spindles. Spinning-disk confocal live-cell imaging of U2OS GFP CENP- E WT and T422A cells (CENP-E in grey and green), infected with AV-H2B-RFP for DNA visualization (blue) and stained for tubulin using SiR-tubulin (red), undergoing the indicated treatments. Time: hour:min.

File name: Supplementary Movie 3
Description: CENP-E T422A stripping from kinetochores. High temporal resolution spinning-disk confocal live-cell imaging of early mitosis U2OS GFP-CENP-E T422A cells (CENP-E T422A in green), infected with AV-H2B-RFP for DNA visualization (magenta). Arrowheads highlight poleward transport of a CENP-E T422A comet. Time: min:sec.

File name: Supplementary Movie 4
Description: CENP-E WT and T422A localization in nocodazole-treated cells entering mitosis. Spinning-disk confocal live-cell imaging of stable U2OS cell lines with inducible expression of GFP-CENP-E WT or T422A (grey and green), infected with AV-H2B- RFP for DNA visualization (blue) and stained for tubulin using SiR-tubulin (red), entering mitosis in presence of nocodazole. Time: hour:min.

File name: Supplementary Movie 5
Description: Mitosis in Spindly-depleted GFP-CENP-E T422A cells. Spinning-disk confocal live-cell imaging of control and Spindly-depleted stable U2OS cell lines with inducible expression of GFP-CENP-E WT or T422A (grey and green), infected with AV- H2B-RFP for DNA visualization (blue) and stained for tubulin using SiR-tubulin (red). Time: hour:min.

File name: Supplementary Movie 6
Description: Mitosis in CENP-E-depleted DHC-GFP cells. Spinning-disk confocal live-cell imaging of HeLa cells stably expressing DHC-GFP (left) and CENP-A- mCherry (center), with SiR-DNA used for DNA staining (right). Time: hour:min.

File name: Supplementary Movie 7
Description: CENP-E localization in metaphase with perturbed MT-flux. Spinning-disk confocal live-cell imaging of stable U2OS cell line with inducible expression of GFP-CENP-E WT (grey and green), infected with AV-H2B-RFP for DNA visualization (blue) and stained for tubulin using SiR-tubulin (red), undergoing the indicated treatments. Time: hour:min.

File name: Supplementary Movie 8
Description: Mitosis in GFP-CENP-E WT and T422A cells upon CENP-E- inhibition by Cmpd-A. Spinning-disk confocal live-cell imaging of stable U2OS cell lines with inducible expression of GFP-

CENP-E WT or T422A (grey and green), infected with AV- H2B-RFP for DNA visualization (blue) and stained for tubulin using SiR-tubulin (red), undergoing the indicated treatments. Time: hour:min.

File name: Supplementary Movie 9
Description: Mitosis in GFP-CENP-E WT and T422A cells upon CENP-E- inhibition. Spinning-disk confocal live-cell imaging of stable U2OS cell lines with inducible expression of GFP-CENP-E WT or T422A (grey and green), infected with AV-H2B-RFP for DNA visualization (blue) and stained for tubulin using SiR-tubulin (red), undergoing the indicated treatments. Time: hour:min.
