# Supplementary material for: CENP-E activation by Aurora A and B controls kinetochore fibrous corona disassembly
Source: Nat Commun. 2023 Sep 1;14:5317. doi: 10.1038/s41467-023-41091-2 (PMC10474297; doi:10.1038/s41467-023-41091-2)
Supplement: Supplementary file 4 — Supplementary Data 1 [file 41467_2023_41091_MOESM4_ESM.docx]

**Supplementary Data 1. Numerical values of the represented data.**

| **Fig. 1c** | **CENP-E dynamics at kinetochore (min)** | **Half-Life ± 95% CI (R^2^)** |
| --- | --- | --- |
|  | WT | 15.23 ± 1.86 (0.97) |
|  | T422A | 5.42 ± 0.63 (0.98) |
| **Fig. 1e** | **Mitotic cells with transient GFP-CENP-E accumulation (%)** | **Mean ± SEM** |
|  | CTRL | 0 ± 0 |
|  | MLN8054 | 57.7 ± 2.2 |
| **Fig. 1g** | **CENP-E/CENP-C intensity ratio at KTs upon the indicated treatments** | **Mean ± SD** |
|  | NOC | 1.6 ± 0.65 |
|  | NOC + ZM447439 | 1.39 ± 0.63 |
|  | STLC | 1 ± 0.37 |
|  | STLC + ZM447439 | 0.29 ± 0.23 |
| **Fig. 2b** | **Stripping velocity (nm/s)** | **Mean ± SD** |
|  |  | 160 ± 25.78 |
| **Fig. 2e** | **GFP intensity at kinetochores (A.U.)** | **Mean ± SD** |
|  | t = 0 min | 1 ± 0.96 |
|  | t = 10 min | 2.83 ± 2.45 |
| **Fig. 2g** | **Corona volume (µm^3^)** | **Mean ± SD** |
|  | WT | 0.093 ± 0.054 |
|  | T422A | 0.083 ± 0.051 |
| **Fig. 2g** | **GFP/CENP-C intensity ratio at kinetochores** | **Mean ± SD** |
|  | WT | 0.97 ± 0.77 |
|  | T422A | 0.96 ± 0.57 |
| **Fig. 3c** | **CENP-E dynamics at kinetochore (min)** | **Half-Life ± 95% CI (R^2^)** |
|  | siCTRL | 7 ± 0.73 (0.98) |
|  | siSPDLY | - |
| **Fig. 3d** | **GFP-CENP-E intensity at the poles (A.U.)** | **Mean ± SD** |
|  | WT + siCTRL | 1.15 ± 1.02 |
|  | T422A + siCTRL | 6.78 ± 4.88 |
|  | WT + siSPDLY | 1.14 ± 1.33 |
|  | T422A + siSPDLY | 0.7 ± 0.59 |
| **Fig. 3e** | **Cells with GFP-CENP-E T422A accumulated at the poles (%)** | **Mean ± SEM** |
|  | siCTRL | 100 ± 0 |
|  | siSPDLY | 4 ± 2 |
|  | siSPDLY + WT | 98 ± 2 |
|  | siSPDLY + SB mut | 33 ± 0 |
| **Fig. 4b** | **DHC dynamics at kinetochore (min)** | **Half-Life ± 95% CI (R^2^)** |
|  | siCTRL | 4.13 ± 0.74 (0.97) |
|  | siCENP-E | 0.81 ± 0.08 (0.99) |
| **Fig. 4e** | **Spindle pole/Total intesity ratio of the indicated proteins in WT/T422A monopoles** | **Mean ± SD** |
|  | GFP_WT | 1 ± 0.18 |
|  | GFP_T422A | 2.13 ± 0.41 |
|  | SPDLY_WT | 1.05 ± 0.21 |
|  | SPDLY_T422A | 1.41 ± 0.31 |
|  | ZW10_WT | 1 ± 0.06 |
|  | ZW10_T422A | 1.27 ± 0.27 |
|  | MAD1_WT | 1 ± 0.16 |
|  | MAD1_T422A | 1.18 ± 0.2 |
|  | BUBR1_WT | 1 ± 0.11 |
|  | BUBR1_T422A | 0.98 ± 0.16 |
| **Fig. 5d** | **Spindle pole/Total intesity ratio of GFP CENP-E WT/T422A** | **Mean ± SD­­­­** |
| ­ | WT_siCTRL_DMSO | 1 ± 0.2 |
|  | WT_siCTRL_GSK923295 | 1.42 ± 0.18 |
|  | WT_siCTRL_Cmpd-A | 1.28 ± 0.19 |
|  | WT_siSPDLY_DMSO | 0.83 ± 0.14 |
|  | WT_siSPDLY_ GSK923295 | 1.33 ± 0.15 |
|  | WT_siSPDLY_ Cmpd-A | 1.03 ± 0.16 |
|  | T422A_siCTRL_DMSO | 1.48 ± 0.25 |
|  | T422A _siCTRL_GSK923295 | 1.43 ± 0.24 |
|  | T422A _siCTRL_Cmpd-A | 1.5 ± 0.26 |
|  | T422A_siSPDLY_DMSO | 1.04 ± 0.17 |
|  | T422A _siSPDLY_ GSK923295 | 1.08 ± 0.18 |
|  | T422A _siSPDLY_ Cmpd-A | 1 ± 0.14 |
| **Fig. S1b** | **Astrin-negative KTs in polar chromosomes per cell (%)** | **Mean ± SD­­­­** |
|  |  | 86.37 ± 18.91 |
| **Fig. S1c** | **Time in mitosis (min)** | **Mean ± SD­­­­** |
|  | WT | 413.5 ± 98.95 |
|  | T422A | 416.6 ± 123.4 |
| **Fig. S5b** | **Cells dividing within 1h (%)** | **Mean ± SEM** |
|  | CENP-E WT | 73.33 ± 4.09 |
|  | CENP-E PP1mut | 76 ± 4.05 |
|  | CENP-E T422A | 4.2 ± 2.1 |
|  | CENP-E T422A PP1mut | 4.3 ± 2.3 |
| **Fig. S6c** | **MT flux (µm/min)** | **Mean ± SD­­­­** |
|  | DMSO | 0.6 ± 0.14 |
|  | 20 nM Noc | 0.18 ± 0.08 |
| **Fig. S6e** | **GFP-CENP-E intensity at x=1 (A.U.)** | **Mean ± SD­­­­** |
|  | GSK923295 | 0.16 ± 0.16 |
|  | GSK923295 + 20 nM Noc | 0.57 ± 0.33 |
|  | Cmpd-A | 0.29 ± 0.29 |
|  | Cmpd-A + 20 nM Noc | 0.25 ± 0.22 |
