# Supplementary material for: CENP-E activation by Aurora A and B controls kinetochore fibrous corona disassembly
Source: Nat Commun. 2023 Sep 1;14:5317. doi: 10.1038/s41467-023-41091-2 (PMC10474297; doi:10.1038/s41467-023-41091-2)
Supplement: Supplementary file 14 — Reporting Summary [file 41467_2023_41091_MOESM14_ESM.pdf]

nature portfolio

Corresponding author(s): Marin Barisic

Last updated by author(s): 21/06/23

# Reporting Summary

Nature Portfolio wishes to improve the reproducibility of the work that we publish. This form provides structure for consistency and transparency in reporting. For further information on Nature Portfolio policies, see our Editorial Policies and the Editorial Policy Checklist.

## Statistics

For all statistical analyses, confirm that the following items are present in the figure legend, table legend, main text, or Methods section.

| n/a | Confirmed | |
|---|---|---|
| ☐ | ☒ | The exact sample size (*n*) for each experimental group/condition, given as a discrete number and unit of measurement |
| ☐ | ☒ | A statement on whether measurements were taken from distinct samples or whether the same sample was measured repeatedly |
| ☐ | ☒ | The statistical test(s) used AND whether they are one- or two-sided<br>*Only common tests should be described solely by name; describe more complex techniques in the Methods section.* |
| ☒ | ☐ | A description of all covariates tested |
| ☐ | ☒ | A description of any assumptions or corrections, such as tests of normality and adjustment for multiple comparisons |
| ☐ | ☒ | A full description of the statistical parameters including central tendency (e.g. means) or other basic estimates (e.g. regression coefficient) AND variation (e.g. standard deviation) or associated estimates of uncertainty (e.g. confidence intervals) |
| ☐ | ☒ | For null hypothesis testing, the test statistic (e.g. *F*, *t*, *r*) with confidence intervals, effect sizes, degrees of freedom and *P* value noted<br>*Give P values as exact values whenever suitable.* |
| ☒ | ☐ | For Bayesian analysis, information on the choice of priors and Markov chain Monte Carlo settings |
| ☒ | ☐ | For hierarchical and complex designs, identification of the appropriate level for tests and full reporting of outcomes |
| ☒ | ☐ | Estimates of effect sizes (e.g. Cohen's *d*, Pearson's *r*), indicating how they were calculated |

*Our web collection on statistics for biologists contains articles on many of the points above.*

## Software and code

Policy information about availability of computer code

| | |
|---|---|
| Data collection | Spinning-disk confocal series and images and widefield images were acquired with SlideBook 6.0.24 Software (3i-Intelligent Imaging Innovations, Inc.). Confocal images were acquired with Zen 2010 B SP1 software (Carl Zeiss, Inc.). Super resolution-SIM images were acquired using Zen Black 3.0 SR FP2 edition software (Carl Zeiss, Inc.). 3D rendering images were generated with Zen Blue 3.2 edition software (Carl Zeiss, Inc.). |
| Data analysis | All images and movies were generated and analyzed using Image J. Kinetochore fluorescence intensities and microtubule flux were analyzed using a custom-written script in MATLAB R2013b (Pereira, A. J., & Maiato, H. (2010). Improved kymography tools and its applications to mitosis. Methods, 51(2), 214-219.). GraphPad Prism 9.1.0 was used for statistical analyses. |

For manuscripts utilizing custom algorithms or software that are central to the research but not yet described in published literature, software must be made available to editors and reviewers. We strongly encourage code deposition in a community repository (e.g. GitHub). See the Nature Portfolio guidelines for submitting code & software for further information.

## Data

Policy information about availability of data

All manuscripts must include a data availability statement. This statement should provide the following information, where applicable:

- Accession codes, unique identifiers, or web links for publicly available datasets
- A description of any restrictions on data availability
- For clinical datasets or third party data, please ensure that the statement adheres to our policy

Quantitative analysis datasets generated in this study will be available as a Source data file. Raw image data generated during this study contain large amount of live-cell videos, confocal image series and super resolution image series and 3D rendering. They are available freely for noncommercial research purposes from the corresponding author on reasonable request.

## Human research participants

Policy information about studies involving human research participants and Sex and Gender in Research.

| | |
|---|---|
| Reporting on sex and gender | n/a |
| Population characteristics | n/a |
| Recruitment | n/a |
| Ethics oversight | n/a |

Note that full information on the approval of the study protocol must also be provided in the manuscript.

# Field-specific reporting

Please select the one below that is the best fit for your research. If you are not sure, read the appropriate sections before making your selection.

☒ Life sciences ☐ Behavioural & social sciences ☐ Ecological, evolutionary & environmental sciences

For a reference copy of the document with all sections, see nature.com/documents/nr-reporting-summary-flat.pdf

# Life sciences study design

All studies must disclose on these points even when the disclosure is negative.

| | |
|---|---|
| Sample size | The sample size in this study is in agreement with the literature in the field. The sample size has been determined by comparing published studies that performed similar type of experiments, e.g. Matkovic et al., Nat Commun 2022 (PMID: 36435852); Castrogiovanni et al., Nat Commun 2022 (PMID: 35948594); Gomes et al., Curr Biol 2022 (PMID: 36057259); Rosas-Salvans et al., Curr Biol 2022 (PMID: 35580605); Sacristan et al., Nat Cell Biol 2018 (PMID: 29915359); Pereira et al., Curr Biol 2018 (PMID: 30415699); Gama et al., J Cell Biol 2018 (PMID: 28320824); Zhang et al., Nat Commun 2017 (PMID: 28604727). |
| Data exclusions | Only cells with undetectable expression of GFP-CENPE WT/T422A were excluded from analysis, as they lacked the signal to be analyzed. No quantifiable data were excluded. |
| Replication | Sample size and number of replicates for each experiment are indicated in the figure legends. |
| Randomization | Cell samples were randomly assigned to a siRNA, drug or control treatment. |
| Blinding | Not applicable because the phenotypes caused by different mutants and treatments were in most cases easily recognizable. Key experiments were performed independently by two different authors. |

# Reporting for specific materials, systems and methods

We require information from authors about some types of materials, experimental systems and methods used in many studies. Here, indicate whether each material, system or method listed is relevant to your study. If you are not sure if a list item applies to your research, read the appropriate section before selecting a response.

## Materials & experimental systems

| n/a | Involved in the study |
|---|---|
| ☐ | ☒ Antibodies |
| ☐ | ☒ Eukaryotic cell lines |
| ☒ | ☐ Palaeontology and archaeology |
| ☒ | ☐ Animals and other organisms |
| ☒ | ☐ Clinical data |
| ☒ | ☐ Dual use research of concern |

## Methods

| n/a | Involved in the study |
|---|---|
| ☒ | ☐ ChIP-seq |
| ☒ | ☐ Flow cytometry |
| ☒ | ☐ MRI-based neuroimaging |

# Antibodies

Antibodies used

Primary antibodies:
- mouse anti-CENP-E Abcam (ab5093), LOT: gr13251-48, diluted 1:500
- rabbit anti-CENP-E Abcam (ab133583), LOT: yk072423ps, diluted 1:500
- rabbit anti-Astrin/MAP126 BETHYL (A301-511A), LOT: A301-511A-1, diluted 1:200
- guinea-pig anti-CENP-C MBL (PD030), LOT:006, diluted 1:2000
- rabbit anti-α-tubulin Abcam (ab15246), LOT: gr3190947-1, diluted 1:500
- mouse anti-α-tubulin Clone B-5-1-2 Sigma (T5168), LOT:00000124629, diluted 1:2000
- mouse anti-MAD1 (clone BB3-8) Milipore (MABE867), LOT: 2931000, diluted 1:200
- goat anti-GFP Rockland (600-101-215), LOT:35052, diluted 1:500
- mouse anti-GFP B-2 Santa Cruz (sc-9996), LOT:BO421, diluted 1:500
- mouse anti-Spindly Abnova (H00054908-M01), LOT:12248-2F4, diluted 1:100
- mouse anti-FLAG-M2 Sigma (f3165), LOT: SLCK5705, diluted 1:1000
- mouse anti-p150 BD trans. Lab (610474), LOT:6295544, diluted 1:1000
- mouse anti-BubR1 (gift from Jakob Nilsson, University of Copenhagen, Denmark), diluted 1:400
- rabbit anti-ZW10 Abcam (ab21582), LOT:gr3176836-1, diluted 1:200
- rabbit anti-CENP-E pT422A (gift from Don Cleveland, University of California San Diego, USA), diluted 1:200
- mouse anti-vinculin Sigma (SAB4200729), LOT:017M4763, diluted 1:5000
- mouse anti-CENP-E Santa Cruz (sc-376685), LOT: J3117, diluted 1:500
- rabbit anti-LIC1 GeneTex (GTX120114), LOT:40373, diluted 1:1000
- mouse anti-MYC (9B11) Cell Signaling (2276), LOT:24, diluted 1:1000

Secondary antibodies:
- Goat anti-Mouse IgG (H+L) Highly Cross-Adsorbed Secondary Antibody, Alexa Fluor 488, 568, 645; Goat anti-Rabbit IgG (H+L) Highly Cross-Adsorbed Secondary Antibody, Alexa Fluo 488, 568, 645; Goat anti-Guinea Pig IgG (H+L) Highly Cross-Adsorbed Secondary Antibody, Alexa Fluor 568, 645; and Donkey anti-Mouse IgG (H+L) Highly Cross-Adsorbed Secondary Antibody, Alexa Fluor 488, 568, 647 (Invitrogen) diluted 1:1000
- Peroxidase AffiniPure Goat Anti-Mouse, goat Anti-Rabbit, donkey anti-goat IgG (H+L)(Jackson ImmunoResearch), diluted 1:10000

Validation

- mouse anti-CENP-E Abcam (ab5093). Suitable for ICC/IF, and WB and used in 35 publications as reported on the manufacturer website (https://www.abcam.com/products/primary-antibodies/cenpe-antibody-1h12-ab5093.html), including Wu et al., Nat Commun 2019 (PMID: 30655516), and Iemura and Tanaka, Nat Commun 2015 (PMID: 25743205). Species reactivity: human.
- rabbit anti-CENP-E Abcam (ab133583). Validated by the manufacturer, suitable for WB and used in 9 publications as reported on the manufacturer website (https://www.abcam.com/products/primary-antibodies/cenpe-antibody-epr45422-ab133583.html), including Steblyanko et al., EMBO J. 2020 (PMID: 33073400), and Gomes et al., Curr Biol 2022 (PMID: 36057259). Species reactivity: human.
- rabbit anti-Astrin/MAP126 BETHYL (A301-511A). Validated by the manufacturer (https://www.fortislife.com/search?query=Astrin%2FMAP126+A301-511A&pageSize=5) and used for IF in Pachis et al., Cell Rep 2019 (PMID: 30784592). Species reactivity: human.
- guinea-pig anti-CENP-C MBL (PD030). Suitable for WB, IP and IF and used in 17 publications as reported on the manufacturer website (https://www.mblbio.com/bio/g/dtl/A/?pcd=PD030#u-pub), including for IF in Nijenhuis et al. Nat Cell Biol 2014 (PMID:25402682), and Etemed et al., Nat Commun 2015 (PMID: 26621779). Species reactivity: human.
- rabbit anti-α-tubulin Abcam (ab15246). Suitable for WB and IHC-P and used in 254 publications as reported on the manufacturer website (https://www.abcam.com/products/primary-antibodies/alpha-tubulin-antibody-microtubule-marker-ab15246.html), including for IF in Sampson et al., EMBO Rep (PMID: 34661367), and Akil et al., Nat Commun 2016 (PMID: 27417143). Species reactivity: human.
- mouse anti-α-tubulin Sigma (T5168). Validated by the manufacturer, suitable for WB and IF and used in 3532 publications as reported on the manufacturer website (https://www.sigmaaldrich.com/DK/en/product/sigma/t5168), including Hut et al., Mol Biol Cell 2003 (PMID: 12802070), and Kalebic et al., Nat Commun 2013 (PMID: 23748901). Species reactivity: mouse, chicken, Chlamydomonas, African green monkey, human, rat, bovine, sea urchin, kangaroo rat.
- mouse anti-MAD1 (clone BB3-8) Milipore. Suitable for WB and IF as reported on the manufacturer website (https://www.merckmillipore.com/DK/en/product/Anti-Mad1-Antibody-clone-BB3-8,MM_NF-MABE867), and used in Screpanti et al., Curr Biol 2011 (PMID: 21353556), and Santaguida et al, EMBO J 2011 (PMID: 21407176). Species reactivity: human.
- goat anti-GFP Rockland (600-101-215). Suitable for WB, IP and IF and used in 340 publications as reported on the manufacturer website (https://www.rockland.com/categories/primary-antibodies/gfp-antibody-600-101-215/), including El Chehadeh et al. (PMID: 35840571), Nat Commun 2022, and Zhang et al., Cell Death Dis 2022 (PMID: 35840557). Species reactivity: jellyfish, all.
- mouse anti-GFP B-2 Santa Cruz (sc-9996). Suitable for WB, IP and IF and used in 3242 publications as reported on the manufacturer website (https://www.scbt.com/p/gfp-antibody-b-2), including Yoshimoto et al., FEBS Open Bio 2023 (PMID: 36680395), and Chauhan et al., Cell Death Discov 2023 (PMID: 36658119). Species reactivity: jellyfish, all.
- mouse anti-Spindly Abnova (H00054908-M01). Suitable for WB as reported on the manufacturer website (https://www.abnova.com/products/products_detail.asp?catalog_id=H00054908-M01) and used for WB and IF in Barisic et al., Mol Biol Cell

2010 (PMID: 20427577). Species reactivity: human.
- mouse anti-FLAG-M2 Sigma (f3165). Suitable for WB, IP and IF and used in 7716 publications as reported on the manufacturer website (https://www.sigmaaldrich.com/DK/en/product/sigma/f3165), including for IP in Anantharaman et al., Nucleic Acids Res 2017 (PMID: 28053121), and for IF in: Liu et al., Virol J. 2016 (PMID: 27630089). Species reactivity: all.
- mouse anti-p150 BD trans. Lab (610474). Suitable for WB, IP and IF and used in 5 publications as reported on the manufacturer website (https://www.bdbiosciences.com/en-eu/products/reagents/microscopy-imaging-reagents/immunofluorescence-reagents/purified-mouse-anti-p150-glued.610474), including for WB and IF in Askham et al., Mol Biol Cell. 2002 (PMID: 12388762), and Tai et al., J Cell Biol 2001 (PMID: 11425878). Species reactivity: human, mouse, rat, dog, chicken.
- rabbit anti-ZW10 Abcam (ab21582). Suitable for WB, IP and IF and used in 22 publications as reported on the manufacturer website (https://www.abcam.com/products/primary-antibodies/zw10-antibody-ab21582.html), including Murillo-Pineda et al., J Cell Biol 2021 (PMID: 33443568), and Allan et al., EMBO J 2020 (PMID: 32202322). Species reactivity: human.
- mouse anti-vinculin Sigma (SAB4200729). Suitable for WB, IP and IF and used in 13 publications as reported on the manufacturer website (https://www.sigmaaldrich.com/DK/en/product/sigma/sab4200729), including Gimona EMBO J 1988 (PMID: 3142762), and Beaudreuil et al., PLoS One 2019 (PMID 31356645). Species reactivity: rabbit, chicken, human, rat, monkey, mouse, canine.
- mouse anti-CENP-E Santa Cruz (sc-376685). Suitable for WB, IP and IF and used in 5 publications as reported on the manufacturer website (https://www.scbt.com/p/cenp-e-antibody-c-5), as well as in She et al., Biochim Biophys Acta Mol Cell Res 2022 (PMID: 35680098), and Liu et al., Cell Cycle 2015 (PMID: 25928583). Species reactivity: mouse, rat and human.
- rabbit anti-LIC1 GeneTex (GTX120114). Suitable for WB, IP and IF and used in 6 publications as reported on the manufacturer website (https://www.genetex.com/Product/Detail/DYNC1LI1-antibody/GTX120114) including Wu et al, Curr Biol 2023 (PMID: 36720222), and Sundararaman et al., Mol Cell 2016 (PMID: 26990993).
- mouse anti-MYC (9B11) Cell Signaling (2276). Suitable for WB, IP and IF and used in 1928 publications as reported on the manufacturer website (https://www.cellsignal.com/products/primary-antibodies/myc-tag-9b11-mouse-mab/2276?_requestid=135724), including Lee et al., Sci Rep 2023 (PMID: 37479820), and Nguyen et al., Nat Commun 2023 (PMID: 37296155). Species reactivity: all.
- mouse anti-BubR1 antibody was described and validated in human cell lines for IF in: Zhang et al., Nat Commun 2016 (PMID: 27457023), and Zhang et al., EMBO J 2019 (PMID: 30782962).
- rabbit anti-CENP-E pT422 antibody was described and validated in human cell lines for IF and WB in: Kim et al., Cell 2010 (PMID: 20691903).

## Eukaryotic cell lines

Policy information about cell lines and Sex and Gender in Research

| | |
|---|---|
| Cell line source(s) | - HeLa (Kyoto): obtained from Danish Cancer Society Research Center's Cell Line Bank<br>- U2OS: gift from Stephan Geley, Innsbruck Medical University, Innsbruck, Austria.<br>- hTERT RPE-1 (ATCC, CRL-4000).<br>- HEK293T: gift from Stephan Geley, Innsbruck Medical University, Innsbruck, Austria.<br>- U2OS Tet-On GFP-CENPE WT and T422A. Generated and described in this paper.<br>- U2OS Tet-On GFP-CENPE WT and T422A CENP-A-mCherry. Generated and described in this paper.<br>- HeLa DHC-GFP (gift from Iain Cheeseman, Whitehead Institute for Biomedical Research, Cambridge, USA).<br>- HeLa DHC-GFP CENP-A mCherry. Generated and described in this paper.<br>- U2OS-PA-GFP/mCherry-α-tubulin cells (gift from R. Medema, Netherlands Cancer Institute - NKI, Amsterdam, Netherlands).<br>- U2OS Tet-On GFP-CENPE W423A and T422A/W423A. Generated and described in this paper. |
| Authentication | The cell lines were not additionally authenticated by the authors. |
| Mycoplasma contamination | Cells were tested regularly for mycoplasma contamination by DAPI staining or PCR. Cell lines tested negative for mycoplasma. |
| Commonly misidentified lines<br>(See ICLAC register) | No commonly misidentified cell lines were used in this paper. |
