# Supplementary figures and images for: CENP-E activation by Aurora A and B controls kinetochore fibrous corona disassembly
Source: Nat Commun. 2023 Sep 1;14:5317. doi: 10.1038/s41467-023-41091-2 (PMC10474297; doi:10.1038/s41467-023-41091-2)

**Fig.1b**

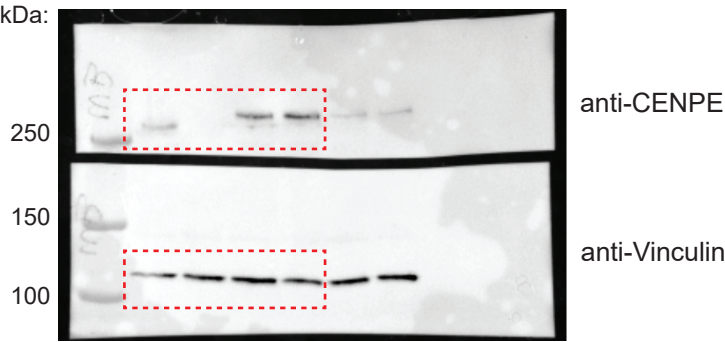

**Fig.3b**

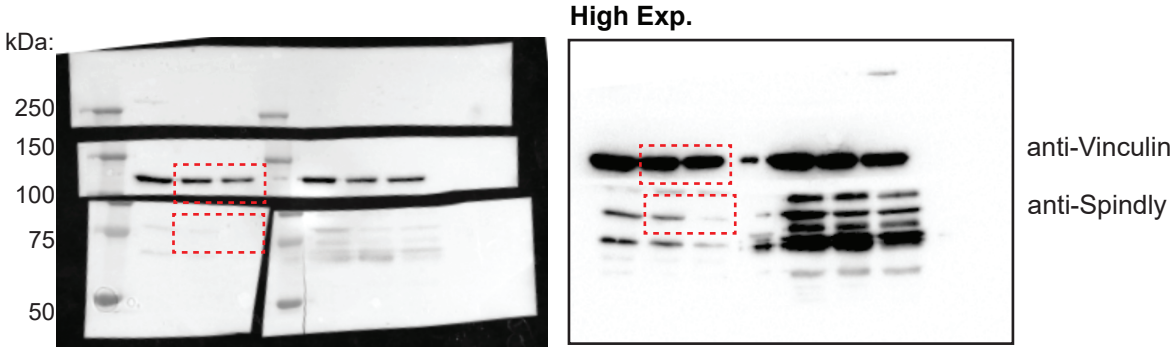

**Fig.3f**

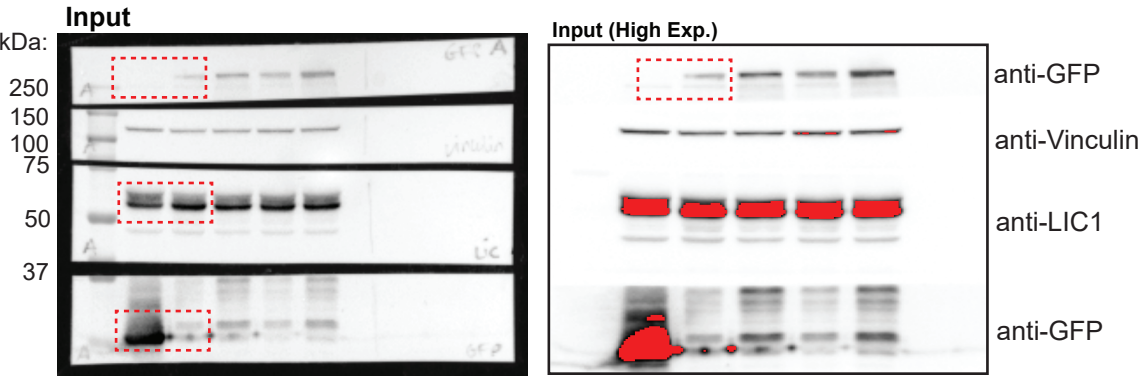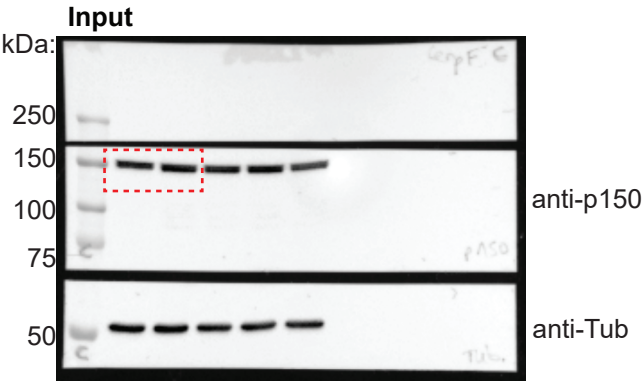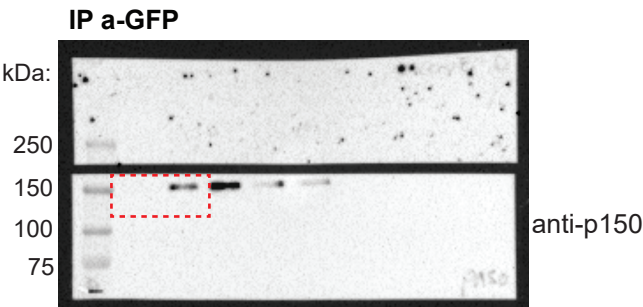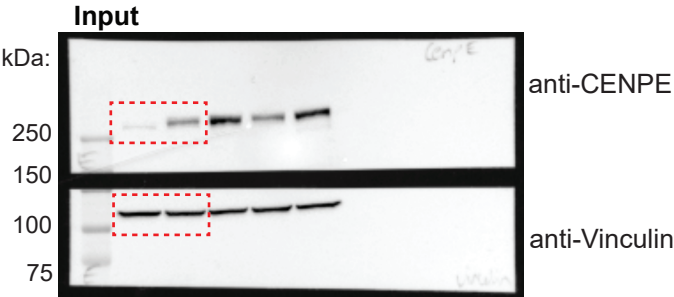

Supplement: Supplementary file 15 — Source Data [file 41467_2023_41091_MOESM15_ESM.zip › Source data/Source Data 1.pdf]

**Fig.3f**

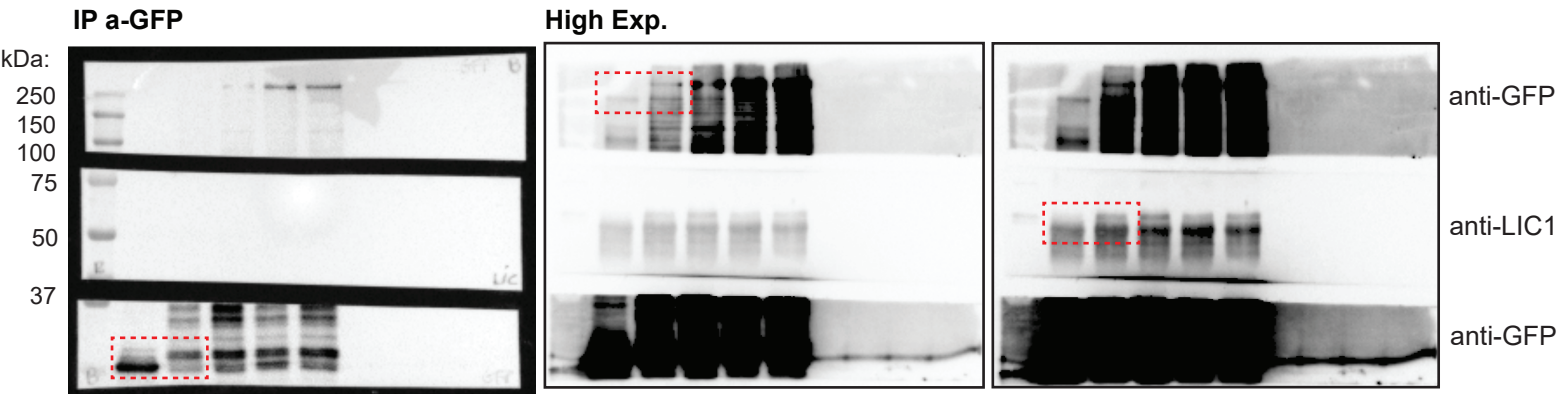

**Fig.5b**

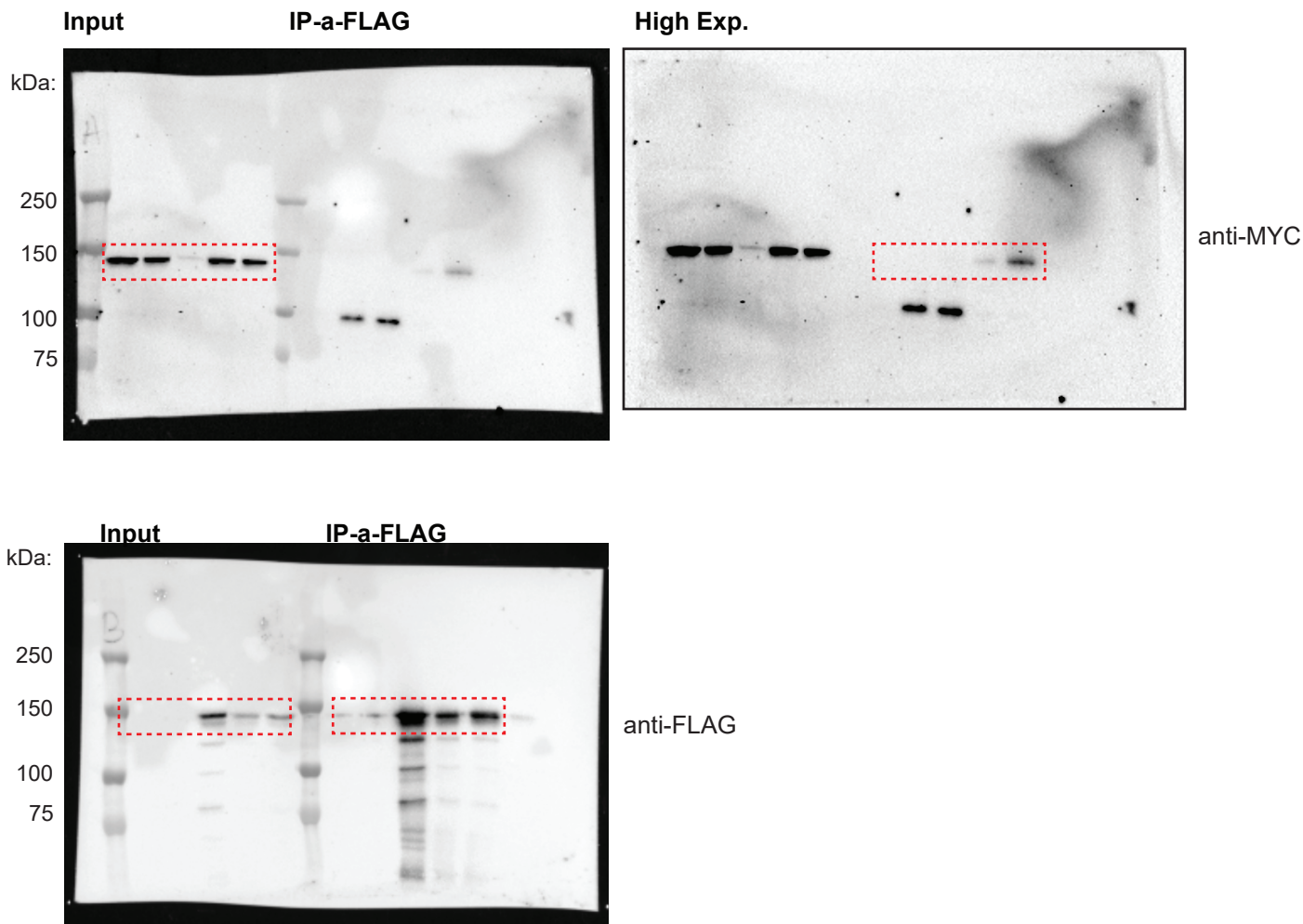

Supplement: Supplementary file 15 — Source Data [file 41467_2023_41091_MOESM15_ESM.zip › Source data/Source Data 2.pdf]
